# Mechanistic Insights into Pulmonary Surfactant Inactivation

**DOI:** 10.1021/acs.langmuir.6c02489

**Published:** 2026-07-05

**Authors:** Maria C. Novaes-Silva, Mariana Rodríguez-Hakim, Jan Vermant

**Affiliations:** † Department of Materials, ETH Zürich, CH-8093 Zürich, Switzerland; ‡ Departamento de Física Fundamental, Universidad Nacional de Educación a Distancia, E28232 Madrid, Spain

## Abstract

Lung surfactant is essential for regulating alveolar surface stresses, reducing the work of breathing, maintaining compliance, and preventing collapse. Under pathological conditions such as acute respiratory distress syndrome (ARDS), this functionality is compromised, yet the underlying physical mechanisms remain incompletely understood. Recent work has shown that surfactant failure cannot be described from surface tension alone, but requires consideration of the interfacial dilatational modulus, which quantifies the ability of the interface to sustain stress under deformation. Mechanical instability arises when this stress-bearing capacity is lost, linking alveolar collapse directly to a reduction in the dilatational modulus. However, this response is typically interpreted in terms of equilibrium adsorption and Gibbs elasticity. Here, we demonstrate instead that it reflects the breakdown of nonequilibrium, microstructure-mediated mechanical surface stresses. By combining freestanding thin-film measurements, cryo-TEM imaging, and dilatational rheology, we isolate the effects of extra compressive stress contributions arising from interfacial microstructure and probe the effects of Albumin and lysophosphatidylcholine (LysoPC) on the clinical surfactant Infasurf. We show that LysoPC-induced structural reorganization disrupts the interfacial architecture, suppressing the development of compressive surface stresses and thereby weakening the mechanical integrity of the interface. These results establish a more subtle link between surfactant microstructure and the interfacial stress response, providing a physically grounded framework for surfactant inactivation and suggesting distinct directions for therapeutic design.

## Graphical abstract

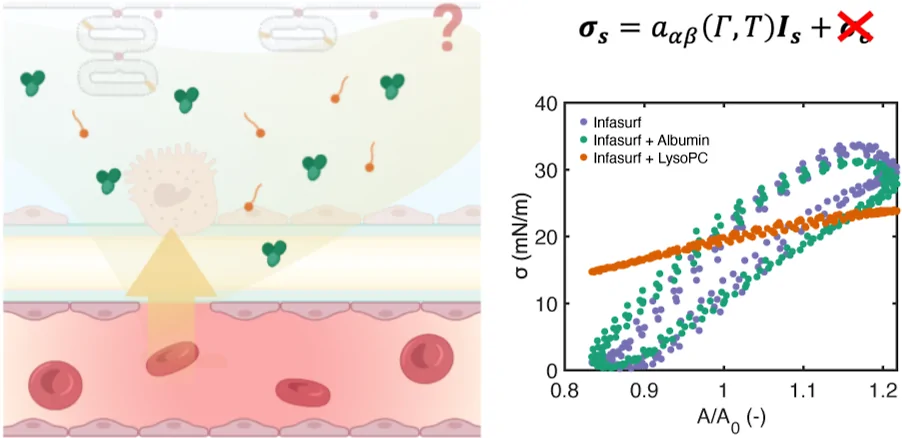

## Introduction

Acute respiratory distress syndrome (ARDS) is a critical form of respiratory failure, associated with high mortality rates.^1–3^ It can be triggered by several infectious and noninfectious causes, including pneumonia and lung trauma.^4–6^ While advances in critical care have reduced the incidence of trauma-related ARDS, new causes have emerged in recent years. Notably, e-cigarette and vaping-associated lung injury was identified in 2018 as a cause of ARDS, especially among young and healthy individuals.^7,8^ More recently, in 2019, ARDS became a major cause of mortality during the COVID-19 pandemic.^7,8^

The pathogenesis of ARDS is complex and not yet fully understood, however, damage to the alveolar-capillary barrier is recognized to play a key role.^4,7^ In healthy lungs, the alveoli and the capillaries are separated by an epithelial-interstitial-endothelial complex, as illustrated in Figure 1.^4^ The capillary endothelium serves as a barrier separating the blood, containing cells and plasma, from the lung interstitium and alveolar airspace. A thin basal membrane is found beneath the endothelial and epithelial cells.^7,9^ The alveolar epithelium is composed of type I cells interspersed with type II cells.^7,9^ The latter synthesize and store lung surfactant as lamellar bodies (LB), which are tightly packed lipid–protein multilamellar assemblies.^10^ These structures are continuously secreted as LB-like aggregates that unravel at the interface, forming an intricate multilayered film.^10,11^ This surfactant film is essential to reduce the alveolar surface stress, decreasing the energy required for breathing, while, at the same time, preventing alveolar collapse.^9,12,13^

**Figure 1:**
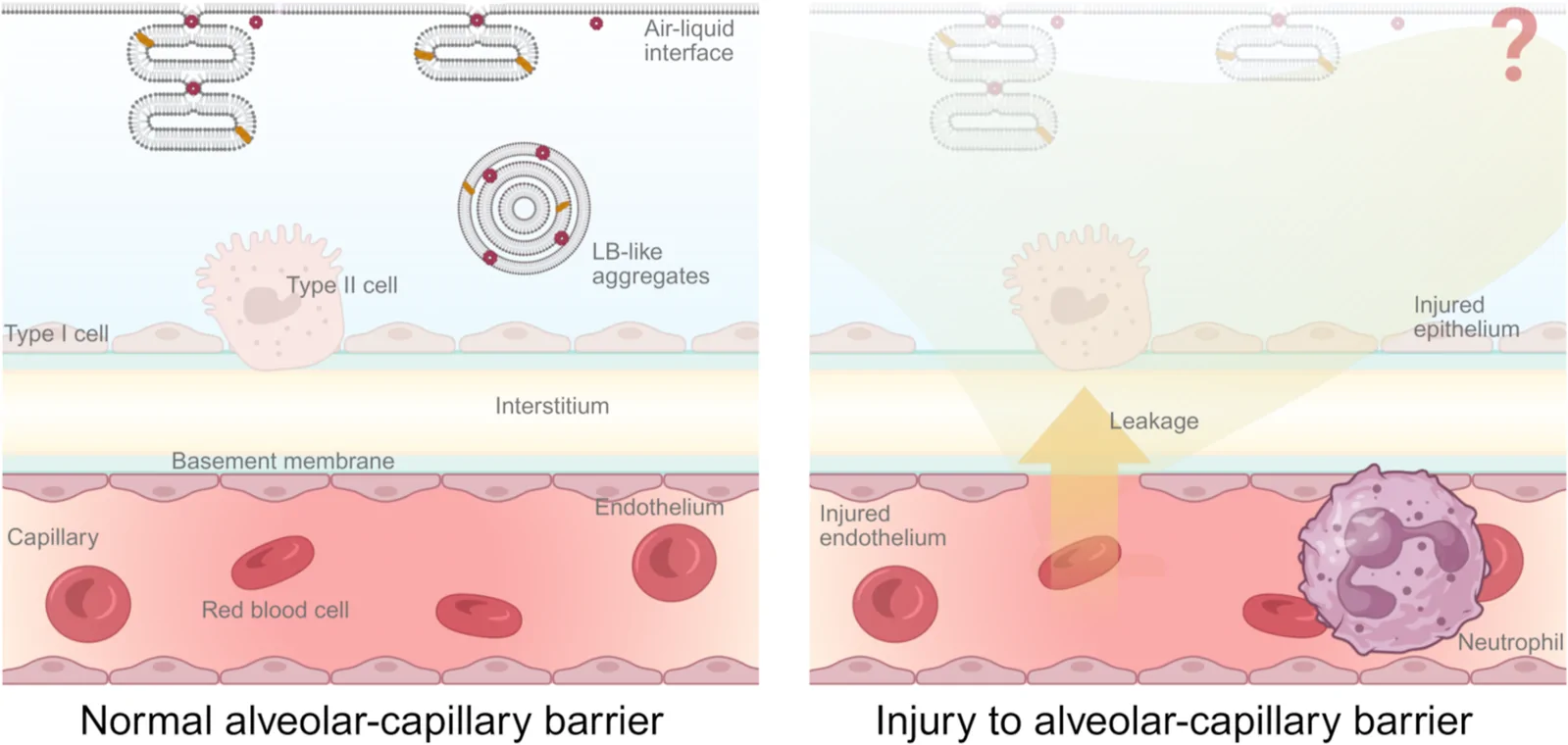
Illustration of a normal and an injured alveolar-capillary barrier. In the normal case, it is composed of alveolar epithelial cells and capillary endothelial cells, which are separated by thin basement membranes. The alveolus epithelium is composed of type I and type II cells. The later produce lung surfactant, which is stored as lamellar bodies (LB). The LBs unravel at the air–liquid interface, forming an interfacial film composed of a monolayer with heterogeneously distributed multilayers. Upon alveolar injury, immune cells (e.g., neutrophils) are recruited. However, their nonspecific action can induce a destruction of the alveolar epithelial-interstitial-endothelial complex, promoting leakage of plasma and associated components into the airspace. Injury to type II cells can impair surfactant production and flooding of plasma content can further inactivate surfactant components. This illustration is for schematic purposes only and is not to scale. Based on the illustration of Bos and Ware^7^ and Castillo-Sánchez et al.^10^ Created in BioRender. Novaes Silva, M. C. (2026) https://BioRender.com/lgkgfpp.

Upon alveolar injury, immune cells, such as neutrophils, are recruited to the inflammation site through the capillaries.^7,14,15^ Activated neutrophils release several components, such as reactive oxygen species, pro-inflammatory cytokines, proteases, and secretory phospholipase A_2_ (sPLA_2_), which can further damage the alveolar-capillary barrier, disrupting both endothelial and epithelium cells.^4,7,14,16,17^ This creates gaps in the epithelial-interstitial-endothelial complex, allowing capillary fluid to leak into the alveolar space.^4,7,10^ The physiological consequences of this injury are severe, including impaired gas exchange, surfactant inactivation leading to alveolar collapse, and decreased lung compliance, which increases the work of breathing.^7^ Due to the complexity of the pathological processes underlying ARDS, no specific treatment currently exists and management remains largely limited to supportive measures.^14,18^ Given the critical role of lung surfactant in proper pulmonary function and the central contribution of its dysfunction in ARDS, it is important to further investigate the mechanisms of surfactant inactivation to guide the development of targeted therapies aimed at restoring its function.

Lung surfactant dysfunction in ARDS is attributed to two primary mechanisms. First, damage to the alveolar epithelium can destroy type II cells, thereby reducing surfactant production.^4,7,19^ Second, plasma leakage introduces components into the alveoli that “inactivate” surfactant.^4,5,7,13,17^ Specifically, serum proteins such as albumin and certain lysolipids, both found at increased concentrations in the alveolar space during ARDS, have been identified as potential inhibitors of surfactant activity,^5,10,20,21^ supported by compositional analyses of bronchoalveolar lavage fluid and in vitro interaction studies.

Different mechanisms have been proposed regarding the effects of serum proteins and lysolipids on the inactivation of lung surfactant. Serum proteins, in particular albumin, have been suggested to compete with lung surfactant components for adsorption at the interface.^5,22–26^ It was claimed that, upon inhalation, albumin, which has a smaller size with a diameter ∼10 nm,^24^ would diffuse faster than the larger micrometer-sized lung surfactant aggregates.^24^ Given the surface-active nature of negatively charged albumin, it adsorbs strongly at the interface and, once adsorbed, creates an electrostatic barrier that hinders lung surfactant adsorption.^5,22–24,26^ However, this hypothesis relies on a diffusion-limited regime and is primarily supported by experiments in which the interface is pre-equilibrated with albumin. Such conditions likely do not reflect the physiological situation, where the interface is initially covered by lung surfactant and both surfactant and albumin already coexist in the subphase, where they interact dynamically. Consequently, this approach limits direct testing of the competitive adsorption hypothesis and has been challenged by subsequent studies.^27,28^

Elevated lysolipid levels have been reported in ARDS and attributed, at least in part, to the activity of sPLA_2_.^13,17,20,21,29^ This enzyme hydrolyzes phospholipids, the main constituents of lung surfactant, into lysolipids (e.g., LysoPC) and fatty acids (e.g., palmitic acid).^13,17,20^ These products have been proposed to penetrate and disrupt interfacial surfactant films, thereby impairing surfactant function.^13,17,29^ Recent studies have further shown that lysolipids can affect the effective dilatational modulus (*K*) of lung surfactant systems.^17,20^ *K* reflects the extent of surface stress variation with respect to a given area strain, providing a measure of the interface’s overall resistance to dilation and compression. These authors have suggested that this modulus may be directly related to alveolar collapse through a Laplace instability criterion, whereby instability occurs when 2*K* < σ,^17,20^ where sigma is the magnitude of the surface stress. However, this interpretation largely emphasizes the role of Gibbs elasticity, in which variations in *K* arise from changes in surface tension caused by area-induced changes in the surface concentration Γ. Within this framework, surface stress variations are attributed primarily to equilibrium adsorption and thermodynamic effects. Such an interpretation, however, neglects the possibility that significant contributions to *K* may also arise from nonequilibrium interfacial rheology, wherein lateral interactions between interfacial constituents and microstructure-dependent mechanical stresses contribute to the surface stress response.^30,31^

In general, the surface stress is a tensorial quantity, and can be written as **σ** = σ_αβ_(Γ, *T*) ***I***_***s***_ + σ_**e**_, where σ_αβ_(Γ, *T*) is the equilibrium thermodynamic surface tension, itself a function of surface concentration Γ and temperature *T*; and σ_**e**_ represents the extra mechanical stresses, capturing the rheological properties. Hence, the effective dilatational modulus contains both an equilibrium thermodynamic contribution arising from σ_αβ_(Γ, *T*) and a rheological contribution associated with the isotropic part of σ_**e**_. The above studies rationalize the Laplace instability criterion solely through changes in σ_αβ_(Γ, *T*), thereby neglecting the interfacial mechanical response due to changes in the interfacial microstructure. As discussed in the seminal work of Meinders and van Vliet,^32^ and consistent with direct measurements of dilatational moduli and drop stability reported, for example, by Rodríguez-Hakim et al.,^31^ isotropic compressive stresses (σ_e_^iso^) can play a significant role in stabilizing shrinking fluid interfaces. In that sense, surfactant inactivation is best viewed not as a surface tension increase per se, but as a mechanically relevant loss of interfacial stress-bearing capacity.

This is particularly relevant for lung surfactant, as interfacial phospholipid films are well-known to exhibit material responses beyond an equilibrium surface tension, including viscoelastic stresses in both shear and dilatation, as demonstrated in experiments and simulations.^33–38^ Such behavior has been documented not only in model phospholipid monolayers but also in more complex lung surfactant-related systems, again under both shear and dilatation,^12,36,39,41^ and originates from the deformation of the interfacial microstructure. This distinction is important because these microstructure-mediated extra stresses, including compressive stresses that oppose collapse, are sensitive to physicochemical conditions and to temperature. Measurements performed at room temperature may therefore probe a different interfacial organization, and hence a different stress response, than under physiological conditions. Consistent with this broader surface-stress picture, Fisher and Squires showed that interfacial shear (extra) stresses during PLA_2_-catalyzed monolayer degradation are highly sensitive to physicochemical conditions.^13^ Accordingly, while PLA_2_-catalyzed degradation of model lung surfactant clearly alters monolayer morphology and mechanics, the mechanisms inferred from simplified, room temperature systems remain to be validated under physiologically relevant conditions and within a comprehensive surface stress framework that considers the role of interfacial rheology and deformation history.

To address this, we adopt a full surface stress framework and investigate the system under physiologically relevant temperatures. In earlier work, we showed that large interfacial expansions, designed to mimic sighs, can induce restructuring of the lung surfactant interface and generate compressive mechanical stresses that offset the overall surface tension, thereby lowering the total stress.^12^ This interpretation is consistent with prior observations and further extends them by demonstrating that surface tension alone is not sufficient to explain interfacial stability: mechanical rigidity and the magnitude of additional mechanical surface stress contributions may be equally important, as has also been recognized for solid-like, e.g. particle-stabilized interfaces.^31,32^

In this work, we revisit surfactant inactivation mechanisms within a full surface stress framework. Using a combination of thin film dynamics, cryo-TEM imaging, and dilatational rheology using a radial through, we investigate the effects of albumin and lysophosphatidylcholine (LysoPC) on lung surfactant properties. Throughout this work, Albumin and LysoPC are introduced at fixed bulk concentrations to represent, respectively, the leakage of serum proteins into the alveolar space and the accumulation of lysolipids resulting from sPLA_2_. Although this constitutes a simplified representation of the in vivo situation, it allows the specific effects of individual inactivating agents on surfactant structure and function to be assessed in a systematic way. In particular, LysoPC is added as a preformed species to the bulk solution, whereas during sPLA_2_ activity it is generated through the degradation of surfactant membranes together with free fatty acids.^13,17,20^ Consequently, the membrane remodeling processes occurring in vivo may differ from those observed here. The structural changes reported in this work should therefore be interpreted as the response of lung surfactant to elevated LysoPC levels rather than as a complete model of phospholipase-mediated degradation.

Our goal is to determine whether competitive adsorption is the primary mechanism driving surfactant inactivation, or whether inactivation agents instead act by modifying interfacial structure thereby altering the surface stress response. This question is particularly relevant in the context of therapeutic strategies for ARDS, where effective treatment depends on understanding the underlying cause of surfactant inactivation, since different mechanisms may require fundamentally different intervention strategies.

## Experimental Section

### Materials

The surfactant replacement Infasurf was provided by ONY Biotech and used as a complete lung surfactant mimic. Fresh solutions diluted in phosphate buffered saline (PBS, Gibco, pH 7.4) were prepared before each experiment at a final concentration of 0.5 mg of phospholipids (PL) per mL. This concentration was chosen based on equilibrium surface tension measurements, where, above 0.4 mg PL/mL, a constant value was reached,^36^ and to match our previous studies.^12,41^

Bovine Serum Albumin (Sigma-Aldrich, ≥98%), hereafter referred as Albumin, was purchased as a powder and dispersed in buffer to a final concentration of 20 mg/mL. Since the competitive adsorption effect of Albumin was shown to be concentration dependent,^29,42^ this value was selected to be above the critical concentration, but still physiologically relevant compared to ARDS levels.^43,44^

LysoPC (Avanti Polar Lipids, 1-palmitoyl-2-hydroxy-*sn*-glycero-3-phosphocholine, 16:0 Lyso PC, >99%) was purchased as a concentrated chloroform solution at 25 mg/mL. The adequate volume was left to evaporate overnight and subsequently vacuumed for 2 h to remove chloroform. The dried lipid film was then rehydrated in buffer to a final concentration of 0.5 mg PL/mL, which corresponds to that of Infasurf. This value was chosen based on in vivo levels measured under ARDS-like conditions^21^ and due to its experimental relevance.^17,20^

For mixtures of Infasurf with Albumin or LysoPC, solutions of Albumin or LysoPC were first prepared at the desired concentration. Infasurf was subsequently added to obtain a final concentration of 0.5 mg/mL, and the mixture was gently homogenized.

### Methods

#### Dynamic Thin Film Balance

A custom-designed borosilicate microfluidic device based on the design of Radke and co-workers^45^ was employed. Details on the geometry and fabrication of this bike-wheel chip can be found elsewhere.^46,47^ In summary, by injecting the relevant solution in the bike-wheel, a freestanding thin liquid film was created, held in place by surface tension. The chip was subsequently placed inside a sealed chamber, whose internal pressure was controlled by an Elveflow MK3+ piezoelectric pressure controller. After sealing, the pressure was gradually increased until interference fringes appeared, indicating the formation of a sufficiently thin liquid film. Once this equilibrium state was reached, two types of experiments were performed. First, the disjoining pressure was evaluated using the equilibrium film method, in which the pressure is increased in discrete steps, allowing the film to reach equilibrium before each subsequent increase.^48,49^ Second, dynamic experiments were conducted to investigate the isotropic drainage of the thin film by applying a larger pressure step of 100 Pa. This more rapid, out of equilibrium drainage protocol not only captures film-thinning dynamics, but also samples the deformation- and microstructure-dependent response of the fluid confined in the gap.^50^ Both types of experiments were conducted at 37 °C and repeated at least three times. To minimize the effect of evaporation, the pressure chamber was partially filled with an excess of buffer solution.

The top view of the freestanding film was imaged using a Nikon Eclipse FN1 fixed-stage upright microscope equipped with a 10× objective and a Hamamatsu ORCA-Flash4.0 CMOS camera. Images were acquired at five frames per second for drainage experiments. A monochromatic wavelength of 508 nm was used and the digital images were converted to thickness via the Sheludko equation^51^

1 $$h=\left(\frac{\lambda }{2\pi n_{f}}\right)\left[m\pi \pm \arcsin\sqrt{\frac{\Delta }{1+4Q\left(1-\Delta \right)/{\left(1-Q\right)}^{2}}}\right]$$

For which $Q={\left[\left(n_{f}-n_{c}\right)/\left(n_{f}+n_{c}\right)\right]}^{2}$ , where *n*_f_ = 1.333 and *n*_c_ = 1 are the refractive indices of the film (water) and the continuous phase (air), respectively,^52^ and Δ = (*I* – *I*_min_)/(*I*_max_ – *I*_min_), where *I* is the intensity of the film, and *I*_max_ and *I*_min_ the maximum and minimum intensities. The integer *m* denotes the order of interference, and *h* represents the equivalent thickness, assuming that the refractive index of the film is equal to that of water. Throughout this work, drainage dynamics are characterized using the film thickness at the center, which is the quantity most commonly reported in thin-film drainage studies.^50,53–56^ This measure provides a robust and reproducible descriptor of film evolution, since spatially averaged or extremal thicknesses are more sensitive to local thickness gradients and heterogeneities that develop during drainage.

#### Adsorption Kinetics

A custom-designed aluminum chamber connected to a circulating water bath was used to hold the experimental solution. The chamber had external dimensions of 9 cm × 9 cm, while the internal cavity, where the solution was placed, measured 6 cm × 6 cm with a height of 1.5 cm. The setup was enclosed in a sealed box to minimize evaporation and maintained at 37 °C. A picture of this setup is provided in the Supporting Information. Surface tension was acquired with a Wilhelmy plate coupled to a balance. To generate a fresh air–liquid interface, the upper surface was aspirated prior to each experiment, removing surface-active components and thus increasing the surface tension. Data were normalized in time such that *t* = 0 s corresponds to the moment at which the surface tension reached its maximum value. Three independent repetitions were performed for each sample.

#### Cryo-TEM

Cryogenic transmission electron microscopy (cryo-TEM) was performed in a Titan Krios FEG (Thermo Fisher Scientific) with a magnification of 6500×, 59,000× or 75,000× and a dose of 50 electrons/Å^2^ for higher magnifications. To prepare the samples, approximately 3 μL of the relevant solution was deposited on a Cu-300 mesh Lacey + 2 nm Copper grid (Quantifoil) and immediately frozen in liquid ethane. Postprocessing to obtain vesicle sizes was performed in MATLAB using the Segment Anything Model (imsegsam), with the confidence score threshold set to 0.8. The area of each detected object was acquired using the regionprops function and a visual inspection was performed to identify and remove falsely detected features.

#### Radial trough

A custom-made radial trough was employed to measure the interfacial dilatational modulus. This setup consists of a circular trough where 350 mL of the relevant solution is placed; 12 motorized fingers surrounded by a SBS elastic band (Vreeberg BV) that encloses the interface, and whose area can be changed by the radial displacement of the fingers; and a Wilhelmy balance coupled to a platinum rod (KSV NIMA) to measure surface stress. Experiments are conducted at 37 °C and repeated twice. A calibration procedure was performed before each experiment, as described in Pepicelli et al.^57^

Oscillatory strain amplitude sweeps were conducted to calculate the effective complex dilatational modulus by the amplitude ratio of the surface stress to the dilatational area strain. A constant frequency of 1 rad/s was used to match the breathing rate, although it is slightly lower than the physiological one (10 breaths/min in our experiments compared to 12–20 breaths/min^58^). However, we believe that this difference does not significantly affect the results. The oscillatory measurements were conducted at a starting area of *A*_0_ = 78.5 cm^2^. Oscillation cycles were composed of an initial interfacial expansion with a prescribed finger displacement, a subsequent recompression to *A*_0_, followed by a compression using the same finger displacement, and a subsequent re-expansion back to *A*_0_, where the finers’ radial position follows a sinusoidal oscillatory motion. Although this motion does not fully capture the complexity of in vivo breathing dynamics, it provides a controlled kinematic deformation that is required for the rheological analysis. Previous studies have shown that the resulting interfacial response is consistent with that obtained under more physiologically relevant breathing-like deformation protocols.^12^ The complete deformation cycle was repeated seven times, with the first two cycles excluded from the analysis. Because the radial trough applies sinusoidal oscillations in the finger position, slight asymmetries arise between expansion and compression. Consistent with the physiological motivation, the interfacial area strain (γ) is defined as the total area change relative to the initial area, i.e., Δ*A*_peak–peak_/*A*_0_.

As one of the objectives of this work is to compare the relative contributions of Gibbs elasticity and interfacial rheology to the overall surface stress response, we compare the thermodynamic modulus, *K*_Π_, with the effective complex dilatational modulus. The thermodynamic modulus was previously measured in our earlier work, where full experimental details can be found.^12^ Briefly, the radial trough was filled with Infasurf at 0.5 mg PL/mL, and the interface was initially set to an area of *A*_0_ = 136.8 cm^2^. The interface was then compressed in nine successive steps, each corresponding to an average compression of approximately 6.5%, resulting in a total compression of 50%. Following each compression step, the surface stress was allowed to relax to its equilibrium value. The relaxation curves were fitted to determine the asymptotic surface stress at infinite time, yielding the equilibrium surface tension for each interfacial area. These equilibrium values were subsequently used to construct a compression isotherm, from which the thermodynamic modulus *K*_Π_ was obtained from the variation of surface pressure with interfacial area strain.

## Results and Discussion

### Evaluating the Role of Competitive Adsorption

Serum proteins and lysolipids, whose concentrations are elevated in the alveolar fluid during ARDS, have both been implicated in lung surfactant inactivation.^5,13,17,20–26,29^ One widely discussed mechanism, particularly for serum proteins such as albumin, is competitive adsorption: because these species diffuse more rapidly than lung surfactant aggregates, they may adsorb first to a clean interface and, in the case of albumin, generate an electrostatic barrier that hinders subsequent lung surfactant adsorption.^5,24,25^ This picture is commonly rationalized in terms of DLVO-type interactions.^24,25^

To experimentally test the competitive adsorption hypothesis, we directly measured the disjoining pressure of freestanding thin films formed in situ from aqueous solutions of Infasurf, Infasurf with Albumin, and Infasurf in the presence of LysoPC. In each case, the air–liquid interfaces are generated during film formation from the corresponding bulk solution. The disjoining pressure represents the excess pressure acting in the normal direction of the film as a result of the overlapping molecular interactions between the two surfaces.^48,59^ This pressure is a function of the film thickness and contains contributions of attractive van der Waals forces (which tend to destabilize the film), repulsive steric forces, and repulsive electrostatic forces (both of which tend to stabilize the film).^59,60^ Although it probes intermolecular forces between the two surfaces of the film instead of a single interface, we propose that variations in the adsorbed species (e.g., lung surfactant vs Albumin), which can seemingly introduce additional electrostatic repulsion, would result in a different disjoining pressure. The results are shown in Figure 2A, together with some interference figures observed upon the first applied pressure step.

**Figure 2:**
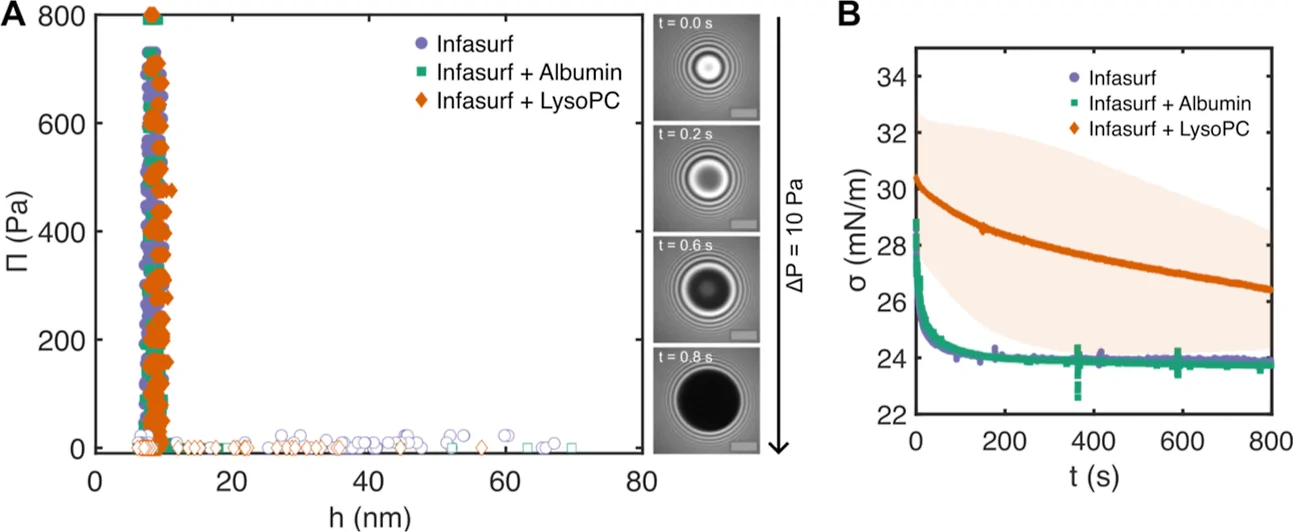
(A) Disjoining pressure (Π) as a function of film thickness (*h*) for Infasurf (purple circles), Infasurf with Albumin (green squares), and Infasurf with LysoPC (orange diamonds), all at 37 °C. Data from three independent repetitions are shown. The empty symbols represent the intermediary film thicknesses obtained during the first pressure step of 10 Pa. The panels on the right-hand side illustrate the top view of the Infasurf with Albumin film upon this first pressure step, showing the transition from an initially thick film at *t* = 0 s (bright film) to a black film at *t* = 0.8 s. The intermediate brightness levels correspond to the thickness values indicated by the empty symbols. Scale bars = 20 μm (lateral dimension). (B) Surface tension (σ) as a function of time (*t*) for Infasurf (purple circles), Infasurf with Albumin (green squares), and Infasurf with LysoPC (orange diamonds). Similarly to the experiments reported in the main text, Infasurf and LysoPC are tested with a concentration of 0.5 mg/mL and Albumin with 20 mg/mL. Markers and shaded regions represent the mean and standard deviation between three repetitions, respectively.

All three samples exhibit indistinguishable disjoining pressure behavior, characterized by a steep Π­(*h*) dependency, within the measurement accuracy of a few nanometers^61^ in film thickness and 10 Pa in pressure step. Even upon the smallest applied pressure step (10 Pa), each film rapidly transitions to a black film, corresponding to an equilibrium minimum thickness.^62^ This initial thickness evolution is indicated by the empty symbols in Figure 2A. The panels on the right show the top view of the Infasurf + Albumin film during this step, where the intensity reflects local thickness, from a bright, thick film at *t* = 0 s to a uniform black film at *t* = 0.8 s. Comparable results have been reported for native and therapeutic lung surfactants.^62–65^

Because all three disjoining pressure isotherms are indistinguishable (within experimental accuracy), the interfacial composition in each case is inferred to be dominated by phospholipids. This observation suggests that neither Albumin nor LysoPC competes effectively for adsorption at the interface under the conditions investigated here. Given that the samples were freshly prepared and the additives coexist with Infasurf in the bulk, any preferential adsorption would be expected to alter the disjoining pressure profile (especially for Albumin).

To further evaluate the role of competitive adsorption, we report the adsorption kinetics of Infasurf, Infasurf with Albumin and Infasurf with LysoPC in Figure 2B. Markers represent the mean surface tension, while shaded regions indicate the corresponding standard deviations. The adsorption kinetics of Infasurf with Albumin closely follow those of pure Infasurf, suggesting that Albumin does not significantly alter the overall adsorption dynamics under the conditions investigated. This observation is consistent with the disjoining pressure results (Figure 2A) and does not provide evidence for sustained competitive adsorption. In contrast, Infasurf in the presence of LysoPC exhibits slower adsorption kinetics compared to pure Infasurf. This difference does not necessarily indicate competitive adsorption and may instead reflect LysoPC-induced modifications of surfactant behavior, as discussed in the following sections. Previous studies have shown that surfactant-associated proteins and lipid organization can significantly influence adsorption kinetics through specific lipid–protein interactions.^10,66^ In this context, structural perturbations induced by LysoPC may alter the pathway of interfacial assembly, thereby affecting the observed adsorption dynamics. Despite the slower approach to equilibrium, Infasurf with LysoPC reaches a surface tension comparable to that of pure Infasurf, although with increased variability in the equilibration time across repetitions.

The present results, which are consistent with previous studies,^27,28^ therefore indicate that competitive adsorption is unlikely to be the dominant surfactant inactivation mechanism. The apparent discrepancy with studies supporting competitive adsorption may arise from differences in experimental protocols. In several previous investigations, Albumin was first allowed to adsorb and equilibrate at the interface before lung surfactant was introduced.^5,22–24,26^ Under such conditions, surfactant adsorption must occur through an existing protein layer, which can enhance inhibitory effects. In contrast, our experiments start from an initially clean interface, with both Albumin and Infasurf present in the bulk and free to adsorb simultaneously. Furthermore, the relatively high concentration of lung surfactant provides a continuous reservoir of surface-active material that can replenish the interface. Together, these factors likely reduce the ability of Albumin to establish a persistent interfacial layer capable of significantly altering the surface stress response. Consistent with this interpretation, the adsorption kinetics measurements reported in Figure 2B show similar adsorption kinetics in the presence and absence of Albumin. It is worth emphasizing that, although our experimental design aims to better reflect physiologically relevant conditions, it remains a simplified representation of the in vivo environment. In particular, the concentrations of lung surfactant and inactivating species were kept constant throughout the experiments, whereas in vivo these concentrations are likely to evolve dynamically during disease progression.^15^

### Effect of Inactivation Products on the Microstructure

While disjoining pressure isotherms are useful for characterizing thin liquid films under quasi-static conditions, and for probing the underlying intermolecular interactions, drainage experiments capture the time-dependent interplay of capillarity, hydrodynamics, and interfacial stresses,^67^ which may be more relevant in the context of breathing. In these experiments, the dynamic drainage behavior of thin liquid films is investigated under a larger pressure step. The resulting evolution of film thickness provides insight not only into the internal structural organization of the confined film, but also into structural rearrangements of the surfactant reservoir in the adjacent bulk phase, including multilayers, micelles, and multilamellar bodies (MLBs), insofar as these structures contribute material to the thinning film. Simple surfactants above the critical micelle concentration have been shown to exhibit stepwise thinning, corresponding to progressive removal of micelles until only a bilayer remains, forming a black film.^56,68^ Similar behavior was previously reported for lung surfactant systems, consistent with the presence of lipid–protein multilayered structures.^11,50^ Considering that LysoPC and Albumin could potentially penetrate lipid membranes and affect these multilayers,^17,29,69^ it seems relevant to investigate the dynamic drainage of the previous three samples. The results are shown in Figure 3. For direct comparison, the drainage curves of all three samples are plotted on the same axes in Figure S2A of the Supporting Information. The individual experimental repetitions for each sample are provided in Figure S2B.

**Figure 3:**
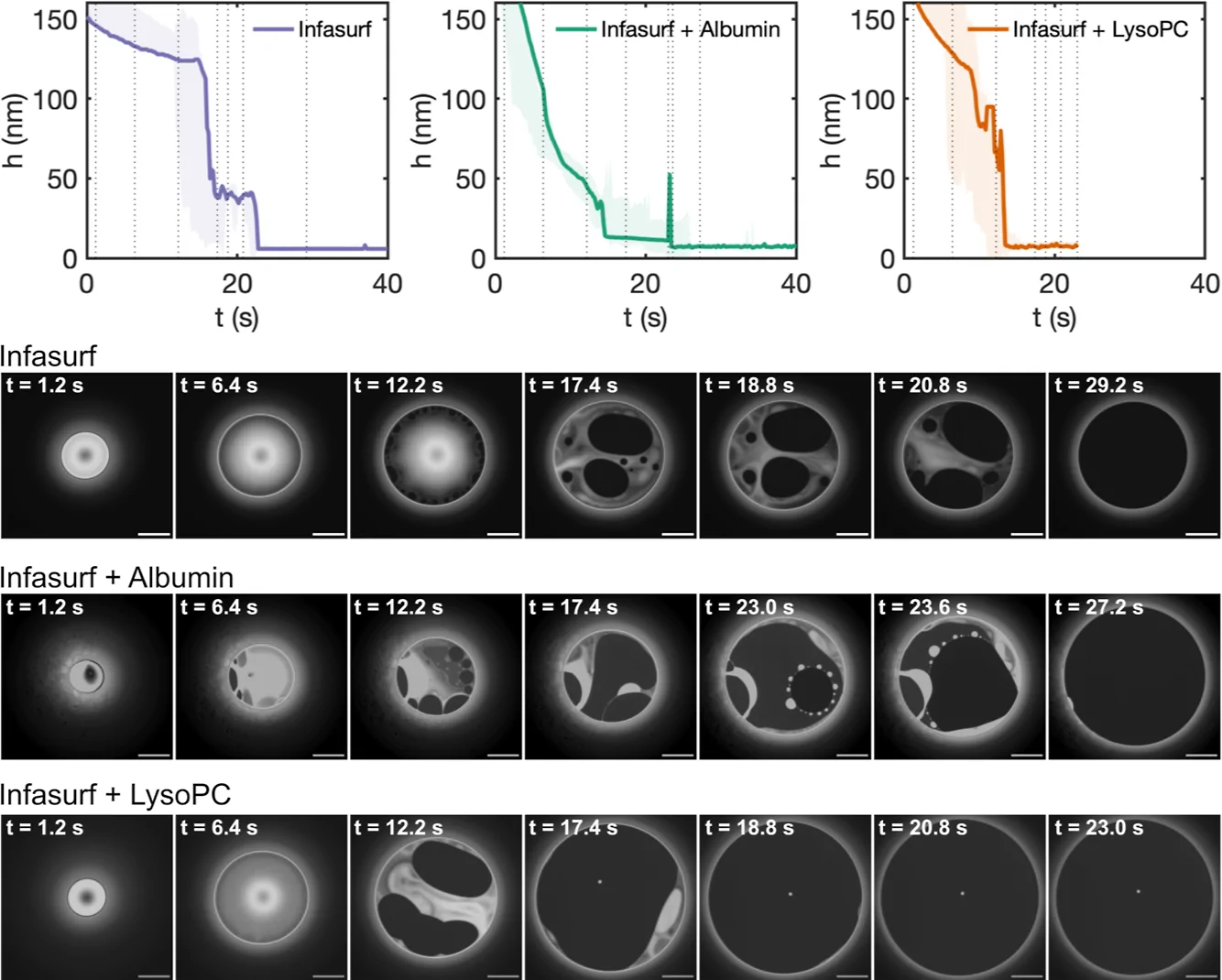
Freestanding film thickness at the center of the film (*h*) as a function of time (*t*) for Infasurf (purple line), Infasurf with Albumin (green line) and Infasurf with LysoPC (orange line) at 37 °C upon a pressure step of 100 Pa. The shaded regions represent the standard deviation across at least three repetitions. Representative images of the top view of the freestanding films during the drainage are shown below the graphs, with the corresponding times indicated by vertical dashed lines. Scale bars are 100 μm (lateral dimension).

The addition of components which lead to surfactant inactivation notably alters the drainage behavior of lung surfactant freestanding films, although some aspects remain in common. Initially, the larger pressure step of 100 Pa generates hydrodynamic and capillary forces,^67^ creating a dimple in the center of the film, which is visually illustrated as the bright region (e.g., *t* = 1.2 s). Subsequently, instabilities take place and lead to a stratification, where different thicknesses coexist (e.g., *t* = 12.2 s). Lastly, a black film is formed, reaching the smallest thickness (e.g., *t* = 23 s for Infasurf + LysoPC). Although all three solutions exhibit similar drainage stages, their thickness evolution differs markedly, particularly in the stepwise thinning. For Infasurf, a distinct thinning step appears around 30 – 40 nm, consistent with the presence of lipid–protein multilayered structures.^50^ This thickness closely matches the multilayer dimensions previously determined by neutron reflectometry.^12,41^ While the present drainage measurements do not allow us to unambiguously distinguish between metastable structural states and kinetic transitions arising during film drainage, the agreement with independently measured multilayer thicknesses suggests that the observed plateau may be associated with a transiently stabilized structural configuration of the interfacial film.

Upon addition of Albumin, this characteristic step remains, suggesting that the bulk structures are largely unaffected. However, an additional thinning step emerges at *h* ∼ 15 nm, comparable to the hydrodynamic diameter of an Albumin molecule.^70,71^ The corresponding images at *t* = 23.0 s and *t* = 23.6 s clearly show these two steps occurring sequentially. Unlike the pure Infasurf multilayers, which coexist with the black film (e.g., *t* = 17.4–20.8 s), Albumin exhibits a distinct transition during step-thinning. This behavior suggests that Albumin molecules coexist with, rather than insert into, the multilayered structures.

In contrast, the addition of LysoPC drastically changes the thickness evolution. The transition from dimple to black film occurs significantly earlier, with only a brief coexistence of thicker features and the black film observed (*t* = 12.2 s). Additionally, the characteristic 30–40 nm step disappears, replaced by a rapid thinning and early transition to a black film. This behavior is illustrated in the lower panels, where the black film completely covers the thin film at *t* = 18.8 s, much earlier than the other two samples. The absence of the multilayer-associated thinning step, together with the rapid black film formation, suggest that LysoPC disrupts or destabilizes the multilayer structure.

To further investigate how these two surfactant inactivation components affect lung surfactant structure in the bulk of the films, we imaged each sample with cryo-TEM. The results are shown in Figure 4.

**Figure 4:**
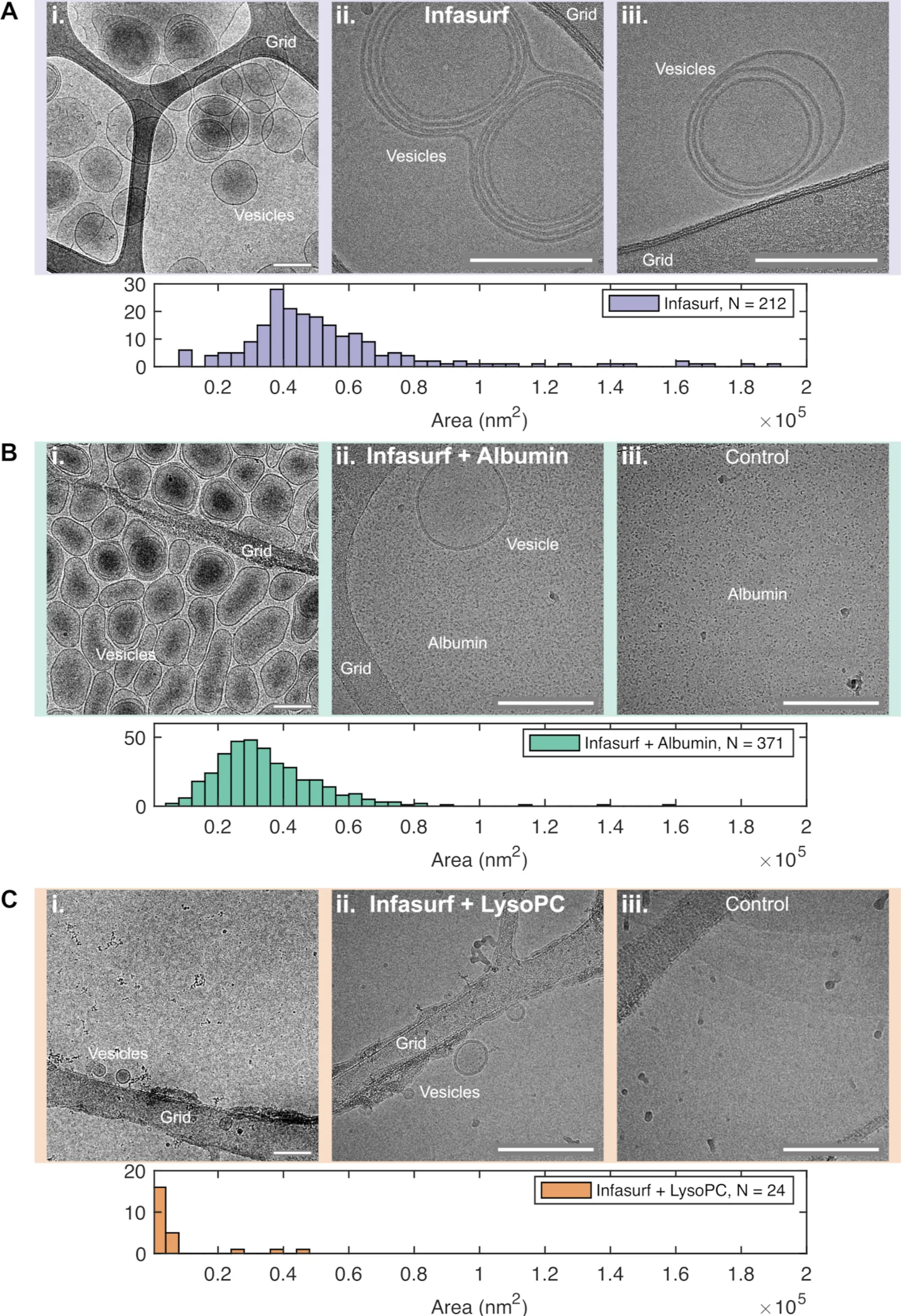
Cryo-TEM images of Infasurf with and without inactivation components: (A) Infasurf bulk structures, showing several uni- and multilamellar vesicles, at 6500× (i) and 75,000× (ii,iii). The graph below illustrates the vesicle area distribution, with the count on the *y*-axis. (B) Infasurf with Albumin bulk structures, showing vesicles similar to pure Infasurf, at 6500× (i) and 59,000× (ii). A control image with only Albumin at 59,000× is shown in (iii). Pure Albumin can be seen as the smaller dark structures. Below, the graph illustrates the vesicle size distribution. (C) Infasurf with LysoPC bulk structures, showing much smaller vesicles than pure Infasurf, at 6500× (i) and 59,000× (ii). A control image at 59,000× with only LysoPC is shown in (iii). No vesicles can be observed. The vesicle area distribution is shown in the graph below. Scale bars are 200 nm.

The bulk structures of Infasurf are shown in Figure 4A, with a lower magnification in (i) and a higher magnification in (ii) and (iii). Large unilamellar and multilamellar vesicles can be observed, similar to those of phospholipids combined with surfactant proteins.^41^ To provide a more quantitative description of the vesicle population, the area distribution is shown below the images, indicating that the majority of vesicles possess a cross-sectional area of 0.4–0.5 × 10^5^ nm^2^. While the analysis includes all vesicles identified across multiple regions of the vitrified grids, only a limited number of grid areas were examined. Consequently, the measured distribution may not fully capture local heterogeneity within the sample. Furthermore, although cryo-vitrification minimizes structural perturbations, preparation-related artifacts, such as local variations in ice thickness and the associated sampling biases, cannot be entirely excluded.

When Albumin is added, similar vesicles can be identified (Figure 4B). At higher magnifications, as shown in Figure 4B­(ii), smaller dark structures are visualized surrounding the vesicle. To verify that these are Albumin molecules, we provide a control image in Figure 4B­(iii), which supports this hypothesis. In the Supporting Information, we provide a size distribution for the Albumin structures obtained with the control cryo-TEM images. The obtained length scale corroborates that Albumin is responsible for the second thinning step at around 15 nm for Infasurf with Albumin in Figure 3 and is in line with previous studies.^70,71^

The area distribution of Infasurf + Albumin is slightly modified compared to that of pure Infasurf, with most vesicles around 0.3 × 10^5^ nm^2^. Despite these minor variations, the overall characteristics are comparable, implying that Albumin does not significantly influence the lung surfactant structure. This is consistent with the conclusions from the freestanding-film drainage results (Figure 3) for Infasurf + Albumin.

On the other hand, when LysoPC is added, the vesicles are significantly different, as shown in Figure 4C. Only a few small unilamellar vesicles can be observed, as also confirmed by the area distribution graph below. Unlike the other two solutions, Infasurf with LysoPC has considerably fewer vesicles and most existing ones have an area of less than 0.1 × 10^5^ nm^2^. Due to the limited number of observed vesicles, the measured distribution in this case is expected to be more susceptible to local sample heterogeneity and potential sampling biases associated with cryo-TEM imaging. Nevertheless, the marked reduction in vesicle abundance relative to the other samples is consistently observed across the analyzed grid regions. The control image with LysoPC alone, shown in Figure 4C­(iii), presents no relevant structures. These observations indicate that LysoPC interacts strongly with lung surfactant vesicles and disrupts their native organization, likely also affecting the multilayered interfacial structures, again consistent with the thin-film drainage results in Figure 3.

This structural disruption is consistent with previously proposed membrane remodeling mechanisms. Upon mixing with lung surfactant, LysoPC interacts with the outer leaflet of surfactant vesicles and becomes selectively incorporated into it. This asymmetric insertion generates lateral tension within the outer leaflet, which cannot be relieved by simple expansion since both leaflets remain coupled. As a result, the bilayer develops a positive spontaneous curvature, promoting membrane budding and fission.^72–75^ Once budding reaches its limit, micellar solubilization may occur.^17,72,76^ This mechanism provides a consistent explanation for the observed reduction in vesicle size and number in the presence of LysoPC. This also might explain the differences in adsorption kinetics shown in Figure 2B.

Overall, Figure 4 demonstrates that Albumin and LysoPC do not inactivate lung surfactant in the same way. Albumin produces only minor changes in the bulk vesicular structure of Infasurf, indicating that the surfactant reservoir remains largely preserved. LysoPC, by contrast, severely disrupts this organization in line with the expected functioning of the lysolipids, yielding fewer and much smaller vesicles and thereby pointing to extensive membrane remodeling and partial solubilization. In combination with the drainage data, these results indicate that LysoPC-driven inactivation is associated with a loss of the multilayered surfactant architecture that underpins the film’s mechanical resilience, whereas Albumin has a much weaker effect on bulk structure.

### The Effect of Altered Structure on the Resulting Surface Stress

The thin-film drainage and cryo-TEM results show that LysoPC markedly alters lung surfactant structure, whereas Albumin appears primarily to coexist with it. In our previous work, we demonstrated that interfacial microstructure plays a central role in regulating the overall surface stress,^12^ beyond the classical surface tension framework. Specifically, we showed that the low surface stresses attained upon sigh-like expansion and recompression arise from structural reorganization of the interface, which generates compressive stresses (a rheological response).^12^ These earlier findings suggest that the distinct structural effects of LysoPC and Albumin should translate into distinct changes in the surface stress. To test this, we measured the effective complex dilatational modulus (|K*|)^77^ at 37 °C of Infasurf in the presence of Albumin or LysoPC and compared these results with those of Infasurf alone, previously reported.^12^ The results are shown in Figure 5, together with a Lissajous plot corresponding to the highest applied strain. The storage modulus (*K*′), loss modulus (*K*″), and phase angle are reported as functions of the interfacial area strain in the Supporting Information. We note that the equilibrium surface tension prior to the dilatational rheology measurements is comparable across all samples.

**Figure 5:**
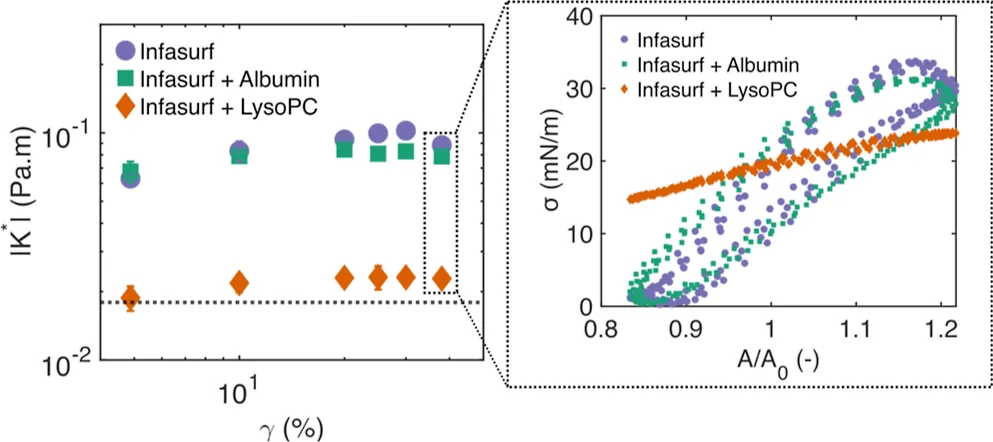
Effective complex dilatational modulus (|K*|) as a function of interfacial area strain (γ) for Infasurf (purple circles, reproduced from Novaes-Silva et al. Copyright 2026 AAAS), Infasurf with Albumin (green squares) and Infasurf with LysoPC (orange diamonds) at 37 °C. The black dashed line represents the purely thermodynamic compressibility of Infasurf, in terms of a modulus (*K*_Π_), reproduced from Novaes-Silva et al.^12^ Copyright 2026 AAAS. The dashed rectangle illustrates the Lissajous plot for a strain of 38%, showing the surface stress evolution (σ) as a function of the interfacial area change (*A*/*A*_0_). The strain amplitude γ corresponds to the maximum variation in area change.

As dynamics matter, a surface stress-based framework is required to properly describe the interfacial properties of lung surfactant.^12^ We emphasize that the rheological contribution is an intrinsic property of the lung surfactant interface and not a consequence of the experimental methodology employed here. Whenever an interfacial deformation is imposed, the measured response corresponds to the total surface stress, which may contain both equilibrium thermodynamic and nonequilibrium rheological contributions. Previous studies have often interpreted changes in the effective dilatational modulus solely in terms of Gibbs elasticity, implicitly attributing the response to variations in surface tension arising from changes in surfactant concentration at the interface. In contrast, the controlled oscillatory deformations imposed in the radial trough allow the complete surface stress response to be quantified through the effective complex dilatational modulus, thereby capturing contributions arising from both equilibrium thermodynamics and microstructure-mediated interfacial rheology. This motivates describing the interface through the surface stress tensor **σ** = σ_αβ_(Γ, *T*) ***I***_***s***_ + σ_**e**_. For isotropic area deformations, this equation simplifies to **σ** = [σ_αβ_(Γ,*T*) + σ_e_^iso^] ***I***_***s***_, and the surface stress becomes a scalar, σ. The extent to which σ varies in response to a given interfacial area strain is captured by the effective complex dilatational modulus |K*|, for which a higher modulus represents a larger σ variation. Since σ has both a thermodynamic (σ_αβ_) and a rheological (σ_e_^iso^) contribution, the response reflected in |K*| will also depend on both. To determine the magnitude of each contribution, the thermodynamic modulus *K*_Π_ can be used as a reference, since it is solely related to σ_αβ_. A brief description of how this modulus is determined is presented in the Methods section and full experimental details can be found elsewhere.^12,57,78^ In Figure 5, *K*_Π_ is shown as the black dashed line. Infasurf’s |K*| is notably higher than *K*_Π_, indicating that compressive mechanical stresses dominate the response. According to our previous work, this mechanical response occurs as a result of an intricate restructuring of the interfacial microstructure. Upon large area variations (i.e., sighs), unsaturated lipids migrate to the multilayers below the interface, enriching the top monolayer with saturated lipids, which can be tightly packed, inducing mechanical stresses.^12^ This suggests that a Gibbs elasticity-based framework is insufficient to fully describe variations in the dilatational modulus, as it accounts only for equilibrium thermodynamic contributions arising from changes in surface concentration and surface tension, not capturing microstructure-mediated surface stress variations.

When Albumin is added to Infasurf, its |K*| remains similar, with only small variations at higher strains (Figure 5). This is consistent with the results of thin film drainage and cryo-TEM which showed no significant structural variation. The Lissajous plot on the right-hand side illustrates the surface stress values as a function of the area variation for the largest applied γ = 38%. As expected, Infasurf with Albumin also reaches physiologically relevant low surface stresses and has an overall similar behavior to that of pure Infasurf. In addition, the equilibrium surface tension value is similar for both (σ­(*A*/*A*_0_ = 1) ∼ 23 mN/m, prior to the oscillation), again arguing against competitive adsorption with lung surfactant. Therefore, the measurements indicate that Albumin does not significantly contribute to the inactivation of lung surfactant under the studied conditions. Nevertheless, some limitations of our approach should be acknowledged. Our experiments were performed at fixed lung surfactant and Albumin concentrations, whereas in vivo both concentrations may vary dynamically with time and disease progression. Furthermore, although the observations provide no evidence for sustained competitive adsorption, we cannot completely exclude the possibility of transient adsorption events occurring on time scales shorter than the time resolution of our measurements. Such events could potentially contribute in a secondary manner to the adsorption dynamics, although they do not appear to dominate the dynamic behavior observed in this work.

On the other hand, adding LysoPC to Infasurf significantly lowers |K*| and the surface-stress response appears to be governed almost entirely by equilibrium thermodynamics, as indicated by |K*|∼ *K*_Π_. This is directly reflected in σ­(*A*/*A*_0_) shown in the Lissajous plot, where a low surface stress can no longer be achieved. We interpret this as a direct consequence of the structural disruption revealed by thin-film drainage and cryo-TEM. Because the bulk vesicles are fewer and markedly smaller, the multilayered structures that normally form beneath the interface upon adsorption are no longer expected to develop. Consequently, the interfacial microstructural rearrangements needed to generate compressive stresses are suppressed. Under these conditions, the interface of Infasurf with LysoPC is expected to behave as a lipid monolayer. Indeed, the variation in surface stress closely resembles that of model phospholipid monolayer.^12,41^ Taken together, these observations emphasize the central role of interfacial microstructure-multilayer structure coupling and compressive stresses in surface stress regulation, further supporting our earlier conclusions.^12,41^

Fisher and Squires also established a direct connection between PLA_2_-induced morphological changes in DPPC monolayers and the corresponding interfacial shear rheology.^13^ During degradation, the formation of palmitic acid and LysoPC markedly modifies the connectivity and thickness of solid-like domains, and these structural changes are accompanied by a reduction in the interfacial shear modulus. While their work was restricted to shear measurements, it is nonetheless consistent with our more general surface-stress framework, which likewise emphasizes that the interfacial mechanical response is controlled by microstructure and its evolution under deformation. Their suggestion of LysoPC as a driver of these morphological changes is now further supported by the observations in the present work.

In this context, there is growing evidence highlighting the importance of the deformation history-dependent nature of lung surfactant interfaces,^12,13,34,79^ with direct implications for surfactant inactivation. Previous studies proposed a Laplace instability criterion for predicting alveolar collapse, whereby 2*K* < σ signals inactivation.^17,20^ These studies demonstrated that LysoPC reduces the dilatational modulus of DPPC and Survanta interfaces, consistent with our observations. However, the decrease in modulus was interpreted exclusively within an equilibrium thermodynamics framework, whereby changes in (*K*) were attributed to Gibbs elasticity and associated with displacement of surfactant components from the interface by LysoPC.^17,20^ The measurements in this work suggest a different interpretation. While LysoPC indeed reduces the dilatational modulus, the combination of cryo-TEM, thin-film drainage, and interfacial rheology indicates that this reduction is accompanied by substantial modifications of the surfactant microstructure. The key insight is therefore not simply that LysoPC lowers *K*, but that variations in *K* can be rationalized through changes in interfacial and bulk microstructure that contribute directly to the surface stress response. Such a connection is difficult to establish within a purely Gibbs elasticity framework, where the modulus is determined solely by a path-independent, equilibrium thermodynamics response.

Instead, we propose a deformation- and history-dependent surface stress framework in which both thermodynamic and rheological contributions determine the measured response. As shown previously, the Gibbs stability criterion can be recast in a similar form, with 2*K* > σ corresponding to stability, but in this formulation *K* reflects the full surface stress response rather than surface tension variations alone.^31,32,80^ Interfacial rheological properties can therefore play a central role in determining stability, with microstructural organization emerging as a key controlling factor. Importantly, this generalized criterion is not restricted to spherical interfaces and can be formulated for arbitrarily curved surfaces through the local curvature field, making it particularly relevant for the complex geometry of alveoli.

Furthermore, although the classical Gibbs criterion retains a similar mathematical form, it describes only equilibrium thermodynamic stability.^31,32,80^ In the alveolar environment, where interfaces are continuously deformed by breathing, such a criterion alone may not fully capture the factors governing long-term stability. The measurements suggest that dynamical effects and interfacial rheology can also play an important role. Extending these concepts to the physiological setting would likely require consideration of additional factors, including breathing patterns, physiologically relevant pressure gradients, realistic alveolar geometry, lung tissue recoil, and the opposing elastic recoil of the chest wall.

In conclusion, the measurements indicate that lung surfactant inactivation arises from the suppression of compressive stresses, resulting directly from structural alterations within the interfacial and subsurface layers. In the case of LysoPC, the observed changes in vesicle morphology and interfacial response are consistent with processes such as vesicle budding and micellar solubilization, which may contribute to persistent modifications of the surfactant microstructure. These findings highlight the importance of considering not only interfacial coverage but also the ability of the surfactant system to generate and sustain compressive stresses.

From a therapeutic perspective, the combined thin film analysis, cryo-TEM and dilatational rheology suggest that both the presence of inactivating species and the restoration of surfactant mechanical function need to be addressed. One possible strategy could involve reducing the concentration of lysolipids and other inflammatory products in the alveolar fluid, for example through compounds that preferentially bind these species. Cyclodextrins represent one potential candidate because their hydrophobic cavity can be tuned to preferentially accommodate single-tailed lipids.^81,82^ In parallel, surfactant replacement therapies may benefit from approaches that restore not only interfacial coverage but also the capacity to generate compressive stresses. Amphiphilic copolymers such as Pluronics have been shown to modify membrane mechanics and enhance stress resilience, making them promising candidates for future investigation.^83^

## Conclusions

This study reframes surfactant inactivation within a surface-stress framework. We show that while some inactivating agents, such as albumin, produce only modest changes in surfactant microstructure and interfacial response, others, such as lysolipids, induce substantial alterations in both interfacial and subsurface organization. These structural changes are accompanied by a marked reduction in the ability of the interface to generate compressive stresses, leading to an interfacial response that becomes increasingly dominated by equilibrium thermodynamic contributions. Our findings therefore suggest that microstructure-mediated rheological stresses play an important role in determining surfactant function and stability. More broadly, the results indicate that therapeutic strategies may benefit from considering not only the restoration of interfacial coverage, but also the removal of inactivating species and the recovery of mechanically active interfacial structures capable of sustaining compressive stresses.

## Supplementary Material



## Notes

Supporting Information contains a zoomed-in version of the disjoining pressure graph, individual repetitions of the drainage experiments, an illustration of the radial trough setup with representative graphs of the analysis, additional data on the phase angle, elastic and viscous contributions related to the dilatational rheology measurements, albumin size distribution, and an illustration of the adsorption kinetics setup (PDF)
