# Supplementary material for: Mechanistic Insights into Pulmonary Surfactant Inactivation
Source: Langmuir. 2026 Jul 5;42(37):27095–106. doi: 10.1021/acs.langmuir.6c02489 (PMC13602926; doi:10.1021/acs.langmuir.6c02489)
Supplement: Supplementary file 1 [file la6c02489_si_001.pdf]

# Supporting Information: Mechanistic insights into pulmonary surfactant inactivation

Maria C. Novaes-Silva,[†] Mariana Rodríguez-Hakim,[‡] and Jan Vermant[*,†]

†*Department of Materials, ETH Zürich, CH-8093 Zürich, Switzerland*

‡*Departamento de Física Fundamental, Universidad Nacional de Educación a Distancia, E28232, Madrid, Spain*

E-mail: jan.vermant@mat.ethz.ch

Number of pages: 9

Number of figures: 7

## Contents

Zoomed-in disjoining pressure . . . . . . . . S2

Drainage repetitions . . . . . . . . S3

Radial trough setup and analysis . . . . . . . . S4-5

Phase angle, elastic and viscous contributions . . . . . . . . S5-6

Albumin size distribution . . . . . . . . S6-8

Chamber for adsorption kinetic studies . . . . . . . . S8

References . . . . . . . . S9

## Zoomed-in disjoining pressure

In the main text, Figure 2A shows the disjoining pressure of Infasurf alone, with Albumin and with LysoPC. In addition to the equilibrium thickness obtained after each pressure step, we also report, as empty symbols, the different thicknesses obtained upon the first pressure step, before reaching the black film. To reduce the thickness scale and confirm the similarity of the disjoining pressure of the three samples, we report in Figure S1 the zoomed-in graph. Each individual repetition is reported with different transparency levels for a clearer comparison.

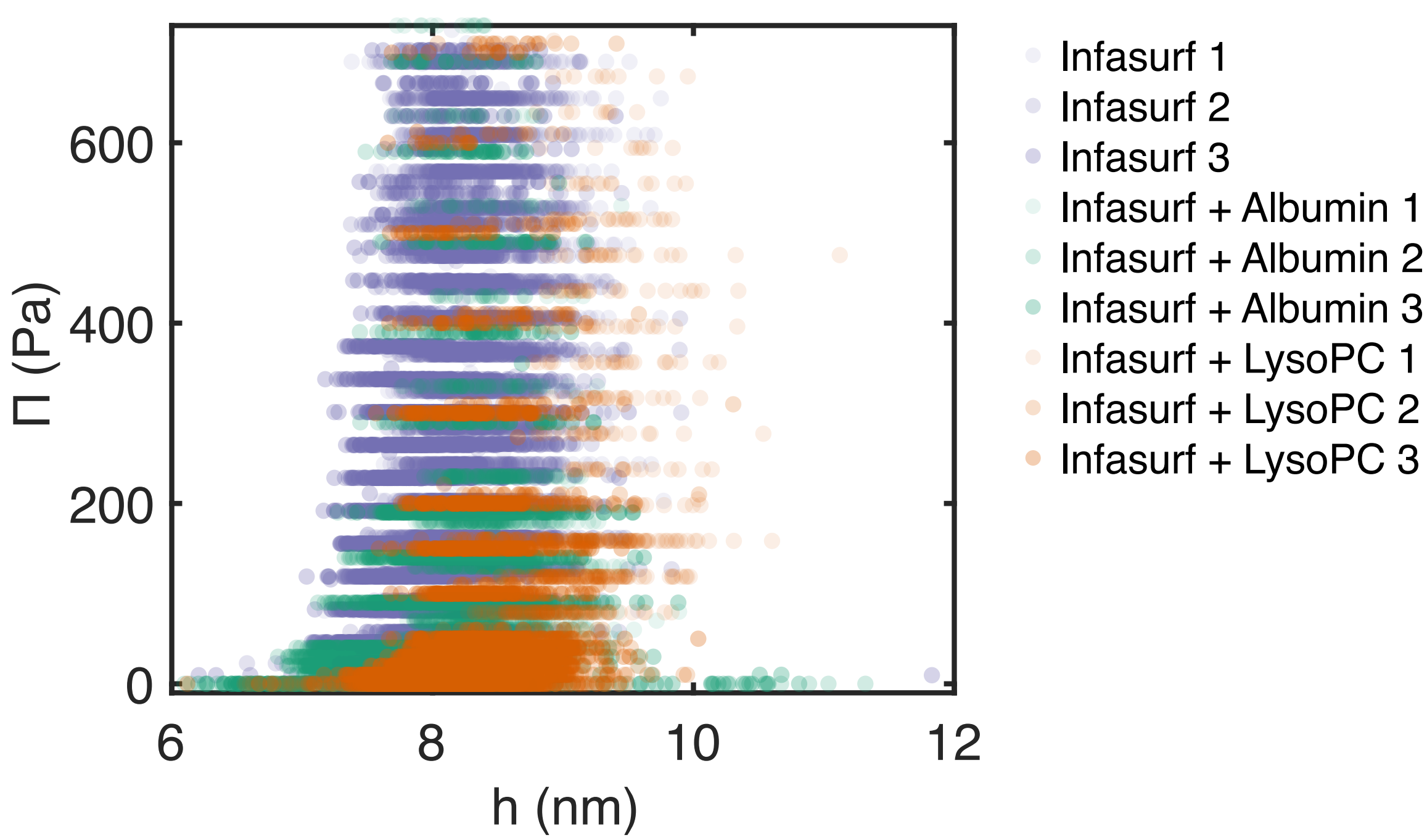


Figure S1: Disjoining pressure (Π) as a function of film thickness ($h$) for Infasurf (purple circles), Infasurf with Albumin (green circles) and Infasurf with LysoPC (orange circles) at 37 °C. All three repetitions are shown in the graph. The differences show the experimental error.

All three systems show a thickness between 7 – 10 nm, with a variation close to the experimental error previously reported.[1] Therefore, we suggest that the differences are not due to the different composition, but arise from experimental variations.

## Drainage repetitions

Figure 3 of the manuscript presents the drainage behavior of the three samples individually. To facilitate a direct comparison, the mean drainage curves for all samples are shown together in Figure S2A. In addition, the individual drainage curves from all experimental repetitions are provided for each sample. As shown, the characteristic stepwise thinning behavior is consistently observed across all repetitions. The primary source of variability arises from differences in the timing of the thinning events, which results in the increased standard deviation observed in the vicinity of the thinning steps.

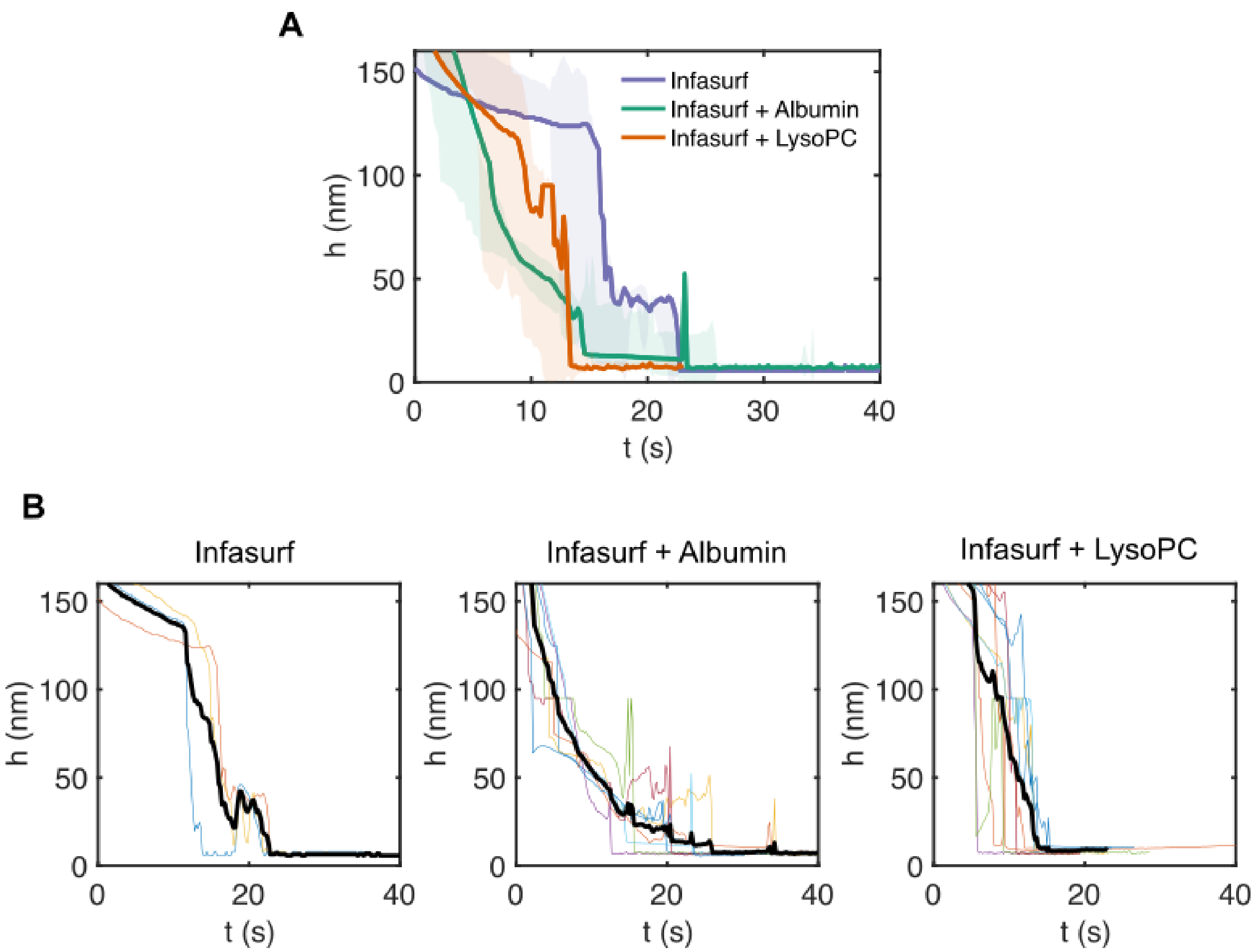


Figure S2: (A) Freestanding film thickness at the center of the film ($h$) as a function of time ($t$) for the three samples: Infasurf (purple line), Infasurf with Albumin (green line) and Infasurf with LysoPC (orange line) at 37 °C upon a pressure step of 100 Pa. (B) Individual drainage curves for each sample, grouped according to the indicated label above each panel.

## Radial trough setup and analysis

An illustration of the radial trough used for dilatational rheology measurements is shown in Figure S3. The setup consists of twelve fingers arranged radially and connected by an elastic band that defines the interfacial area. The motion of the fingers is controlled by a stepper motor, allowing the interface to undergo prescribed expansions and compressions. A Wilhelmy rod positioned at the center of the trough continuously measures the surface stress response, while a thermocouple monitors the interfacial temperature throughout the experiment. The lower panels illustrate the raw data for the oscillations of a representative amplitude. The motor position (circular markers) follows a sinusoidal trajectory, producing a controlled oscillatory deformation of the interface, with only minor deviations from the ideal sinusoidal motion (red line). The resulting surface pressure response is also periodic but may exhibit both amplitude and phase differences relative to the imposed deformation. By fitting the motor position and surface pressure signals to sinusoidal functions, the amplitude and phase shift of the interfacial response can be quantified. These quantities are subsequently used to determine the complex dilatational modulus and thereby characterize the elastic and viscous contributions to the interfacial rheology. As shown by the surface pressure data, vibrations generated by the motion of the fingers contribute only a minor level of noise, which is negligible compared with the measured signal. The motor displacement is converted into interfacial area variations using the known initial area, $A_0 = 78.5\ \text{cm}^2$, and the motor calibration factor (one step is 0.124 $\mu$m). The interfacial area strain is then defined as the peak-to-peak area variation normalized by the initial area, $\gamma = \Delta A_{\text{peak-to-peak}}/A_0$. To illustrate the calculated area at the largest applied strain, its variation with time is reported in Figure S4, together with the corresponding sinusoidal fit.

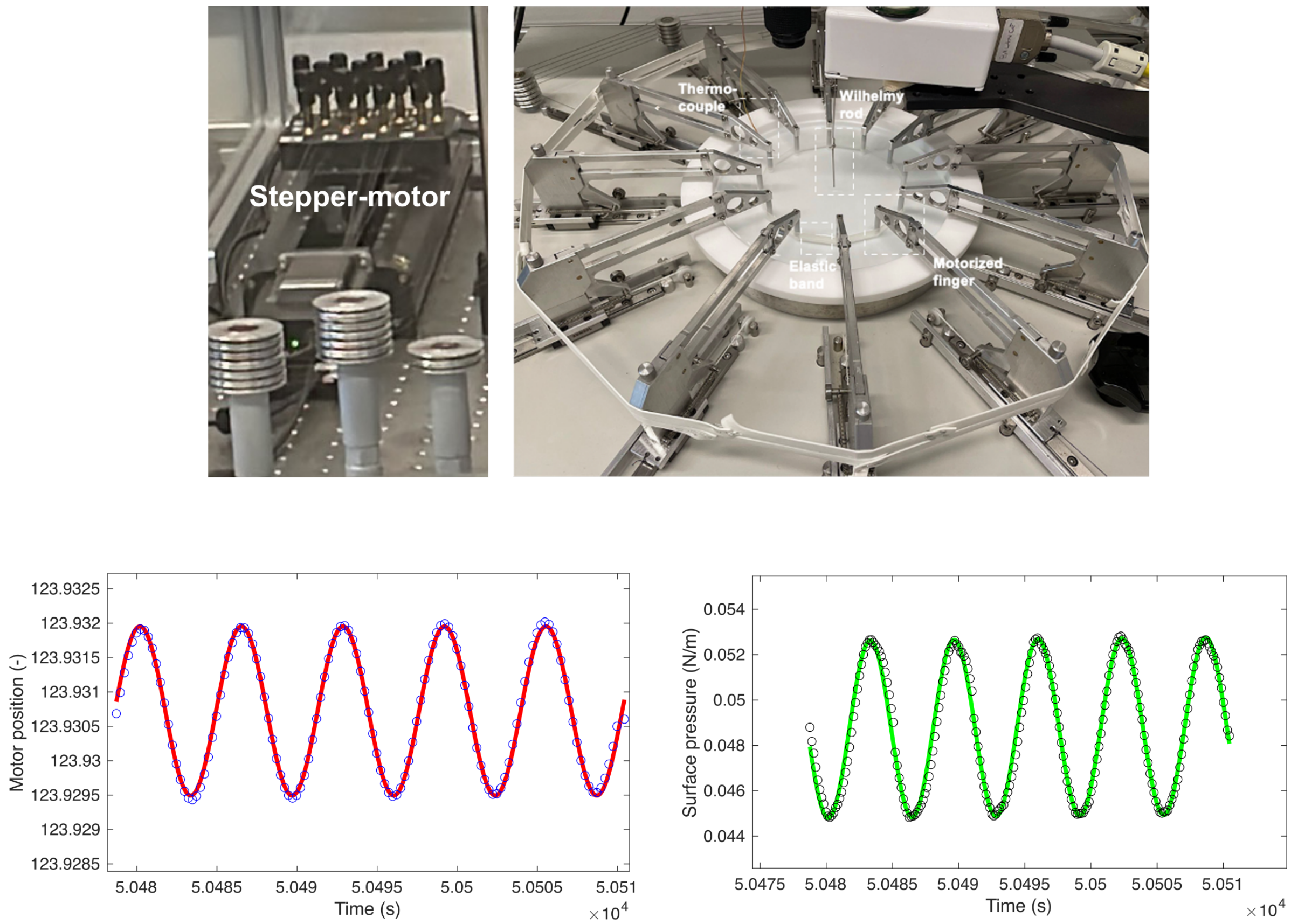


Figure S3: Illustration of the radial trough setup. The upper left panel shows the stepper motor that controls the motion of the twelve fingers. The upper right panel shows the radial trough containing the sample solution, with the twelve motorized fingers enclosed by an elastic band that defines the interfacial area. A Wilhelmy rod positioned at the center of the trough is used to measure the surface stress, while a thermocouple placed at the interface monitors the temperature. The lower panels show the motor position and surface pressure as functions of time (circular markers). Here, the surface pressure is defined as the surface tension of the clean interface ($\sim 70$ mN/m) minus the measured surface stress. The solid red and green curves represent sinusoidal fits to the motor position and surface pressure data, respectively.

## Phase angle, elastic and viscous contributions

To further illustrate the viscoelastic response of the three samples, the storage modulus ($K'$), loss modulus ($K''$), and phase angle are reported as functions of interfacial area strain ($\gamma$) in Figure S5. For all samples and across the entire strain range investigated, the interfacial response remains predominantly elastic, as evidenced by $K' > K''$. Nevertheless, the relative viscous contribution increases with increasing strain, reflected by the gradual increase in the

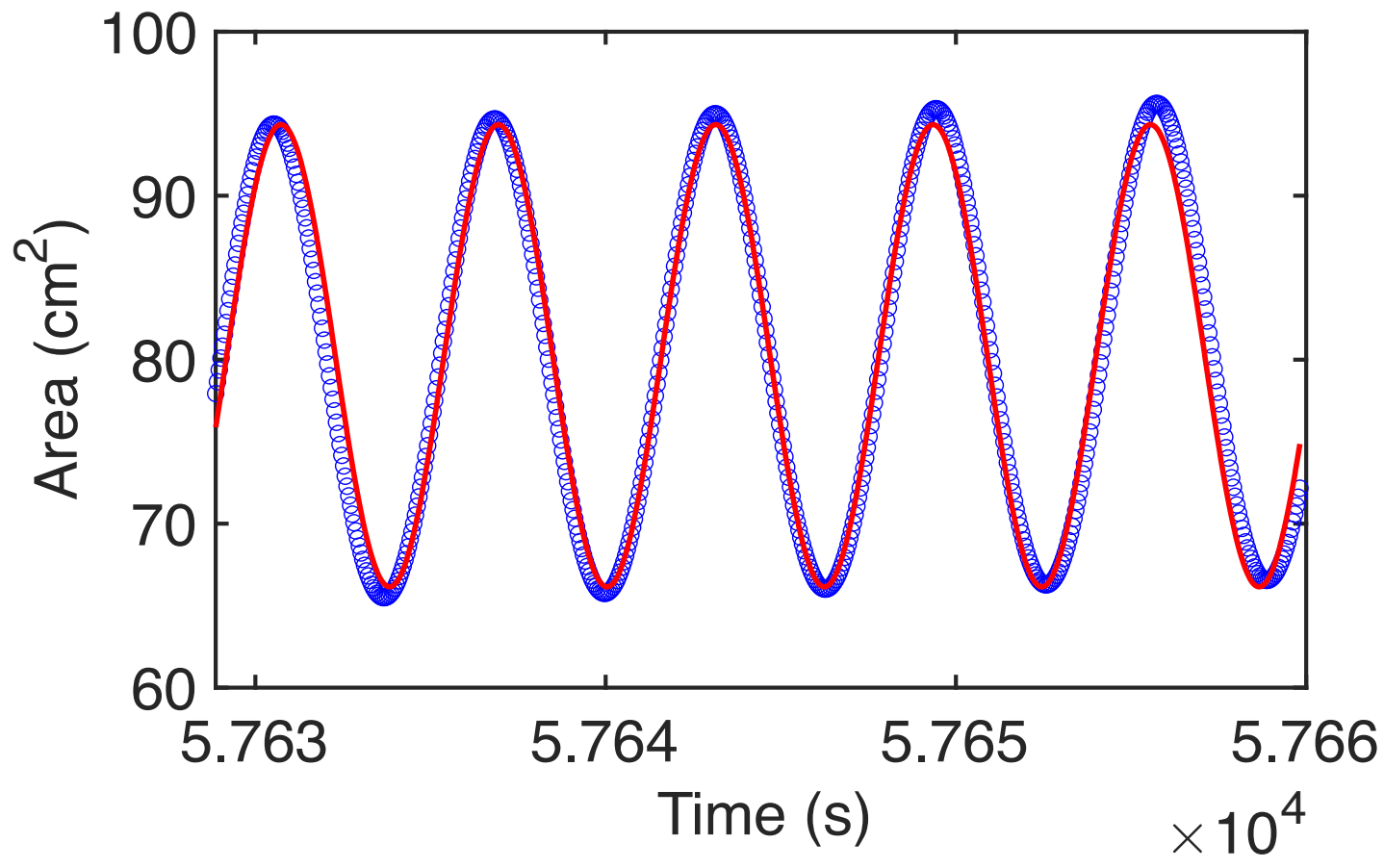


Figure S4: Calculated area as a function of time for the largest strain applied. The solid red line represent the sinusoidal fit.

phase angle.

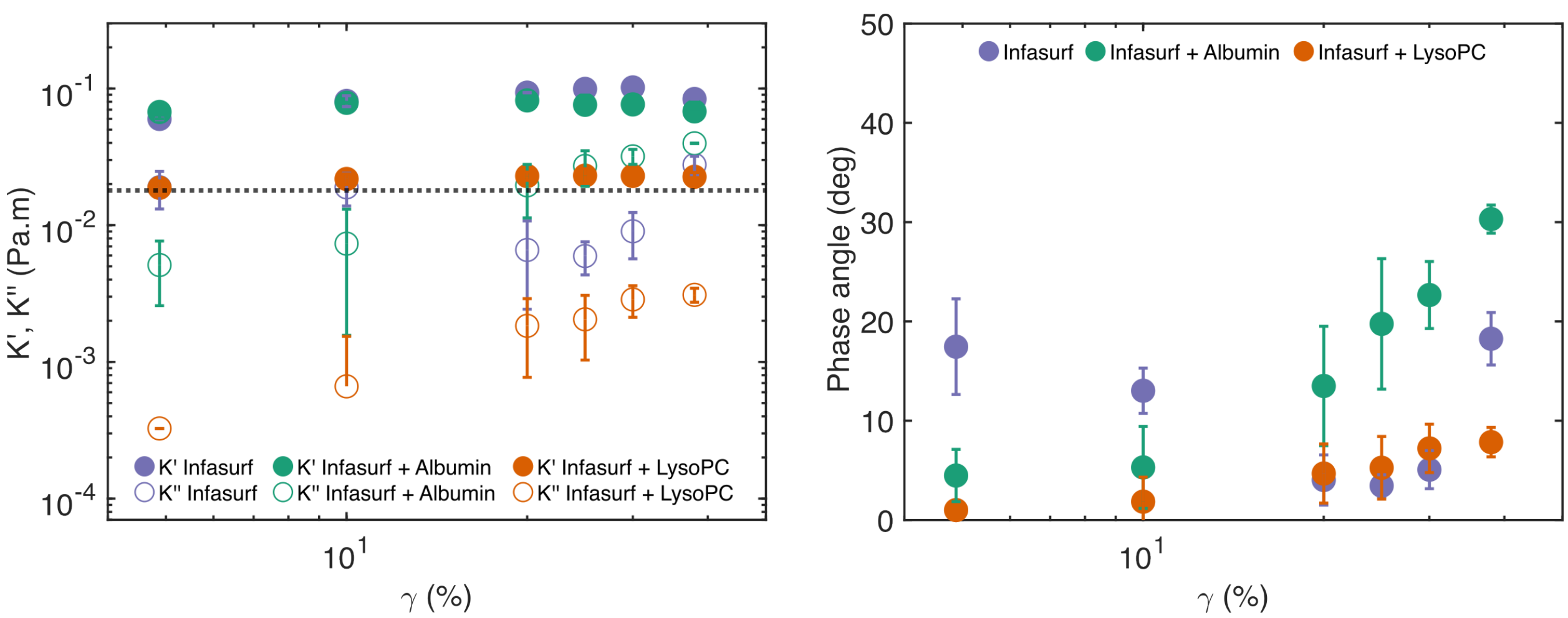


Figure S5: On the left panel, dilatational moduli, elastic $K'$ (filled markers) and viscous $K''$ (empty markers) as a function of interfacial area strain ($\gamma$) for Infasurf (purple), Infasurf with Albumin (green) and Infasurf with LysoPC (orange). On the right panel, phase angle as a function of interfacial area strain ($\gamma$) for Infasurf (purple), Infasurf with Albumin (green) and Infasurf with LysoPC (orange).

# Albumin size distribution

Albumin protein sizes were determined through two cryo-TEM control images. The post-processing steps consisted of filtering the images with a 2D Gaussian smoothing kernel with

standard deviation of 10; enhancing the contrast of the smoothed image by using a contrast-limited adaptive histogram equalization; binarizing the image through an Otsu-based threshold;[2] removing components with less than 500 pixels; and performing a morphological closing (dilation followed by erosion) of the image using a circular object with 5 pixels of radius. Then, using the same procedure as that for the vesicle area, we obtained the major and minor axes. The results are reported in Figure S6.

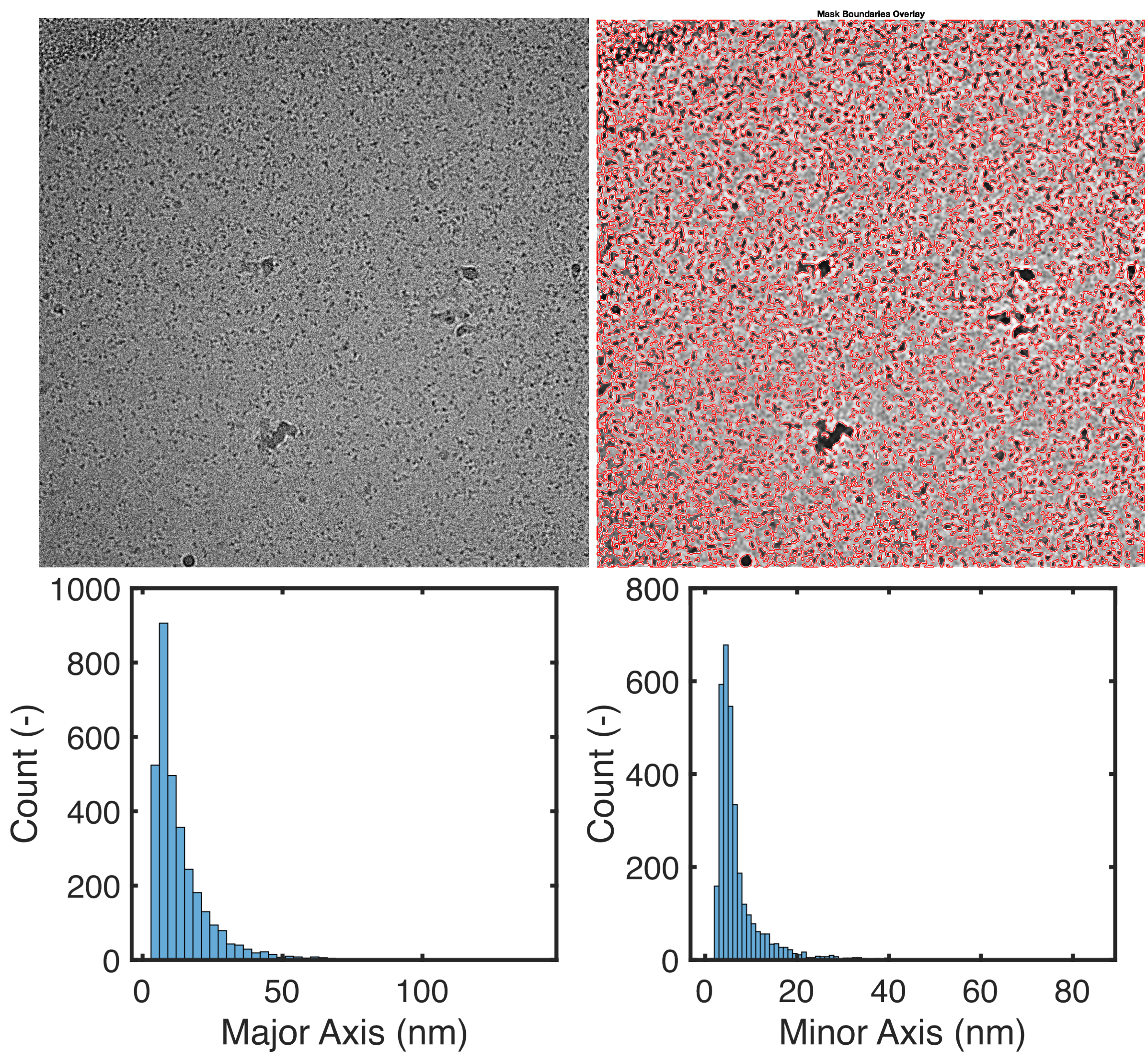


Figure S6: The upper part illustrates the raw control image on the left and the post-processed image with the overlayed mask of the binary detected features on the right. Albumin size distribution is shown on the bottom, showing on the left-hand side the major axis and, on the right-hand side, the minor axis, both in nm.

It should be noted that, as can be seen in Figure 4B, some larger features, potentially due to ethane contamination, are also present in the cryo-TEM control image. This could explain the larger sizes detected. The median size for the major axis was 9.9 nm with a median absolute deviation of 3.9 nm. For the minor axis, the median value was 5.3 nm with a median absolute deviation of 1.5 nm. These dimensions match previous values reported in the literature,[3,4] and are of the same order of magnitude as the ones of the second step-drainage of Infasurf with Albumin in Figure 3.

## Chamber for adsorption kinetic studies

An illustration of the chamber used for the adsorption kinetic studies is shown in Figure S7. The top view is shown on the left, and a side view of the chamber with the enclosing box is shown on the right. A circulating water bath inside the chamber enables temperature control.

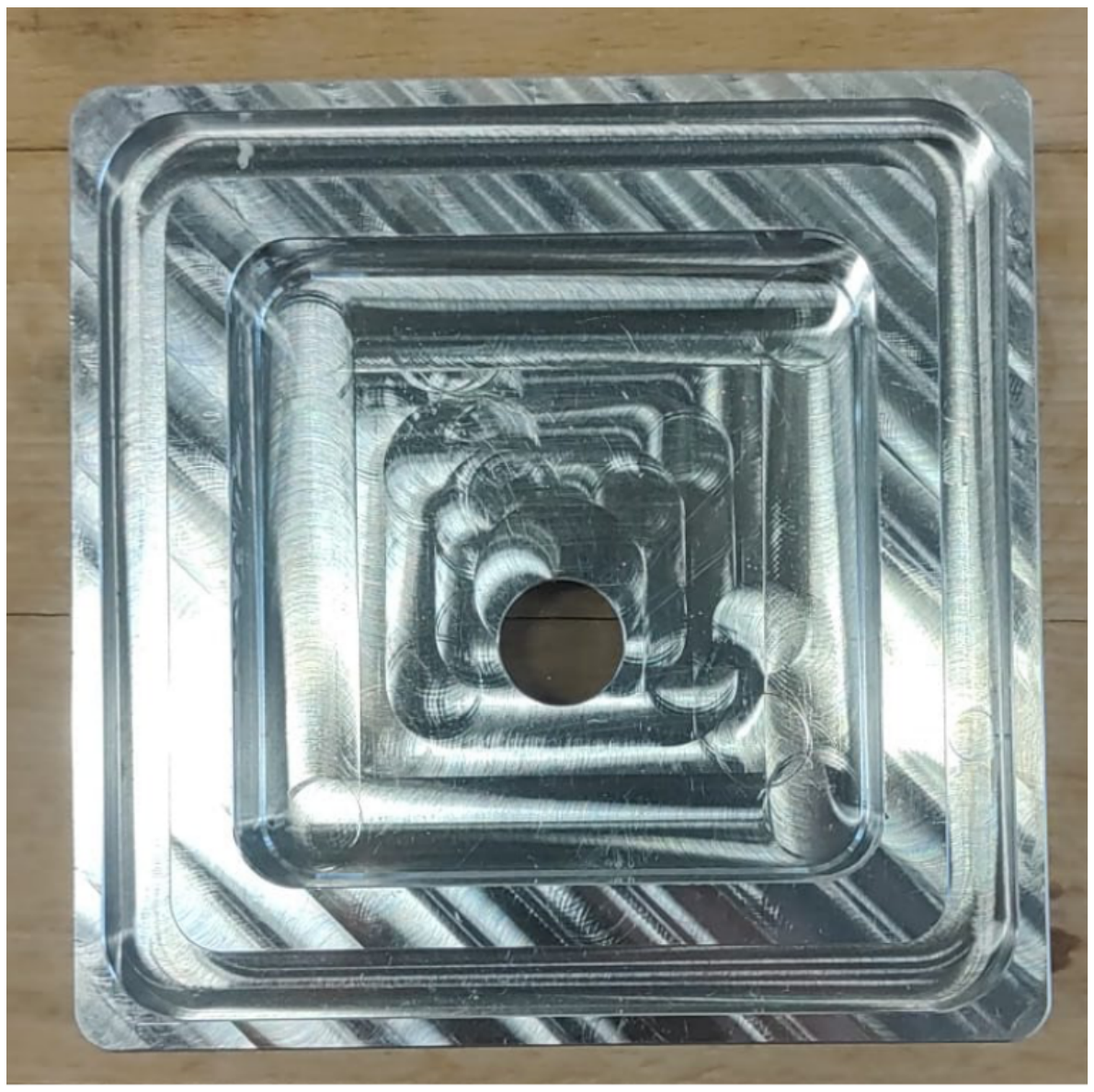

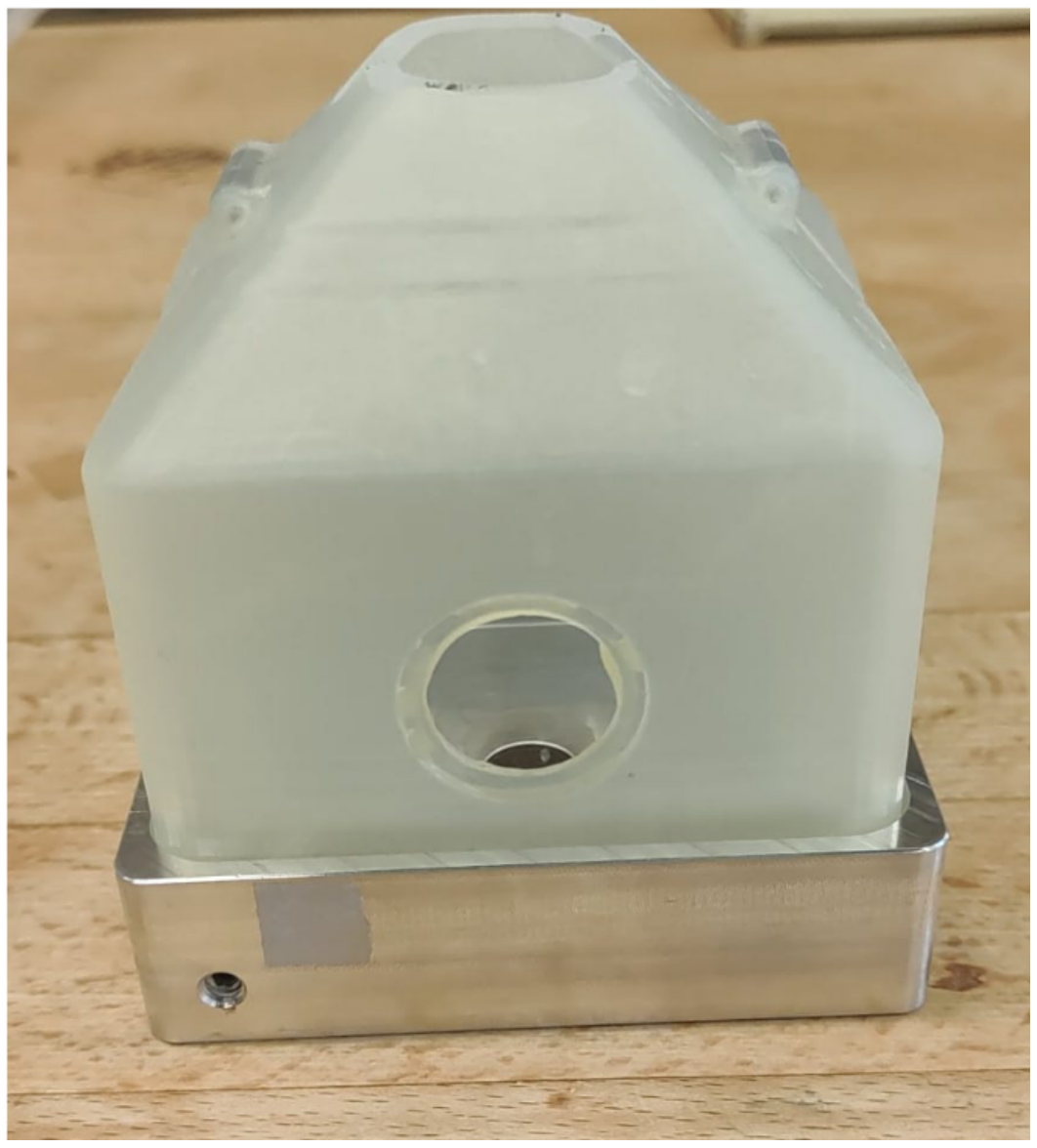

Figure S7: Chamber used for adsorption kinetic studies. On the left-hand side, the top view of the temperature controlled-chamber. On the right, the side view of the chamber with the enclosing box.

# References


(1) Chatzigiannakis, E.; Veenstra, P.; ten Bosch, D.; Vermant, J. Mimicking coalescence using a pressure-controlled dynamic thin film balance. *Soft Matter* **2020**, *16*, 9410.

(2) Otsu, N. A Threshold Selection Method from Gray-Level Histograms. *IEEE Trans. Syst. Man Cybern.* **1979**, *9*, 62–66.

(3) Matsarskaia, O.; Bühl, L.; Beck, C.; Grimaldo, M.; Schweins, R.; Zhang, F.; Seydel, T.; Schreiber, F.; Roosen-Runge, F. Evolution of the structure and dynamics of bovine serum albumin induced by thermal denaturation. *Phys. Chem. Chem. Phys.* **2020**, *22*, 18507–18517.

(4) Jachimska, B.; Pajor, A. Physico-chemical characterization of bovine serum albumin in solution and as deposited on surfaces. *Bioelectrochemistry* **2012**, *87*, 138–146, International Symposium on Bioelectrochemistry and Bioenergetics, 21st BES 2011.
